# Identification of Autophagy-Related Prognostic Signature and Analysis of Immune Cell Infiltration in Low-Grade Gliomas

**DOI:** 10.1155/2021/7918693

**Published:** 2021-11-08

**Authors:** Qingli Quan, Xinxin Xiong, Shanyun Wu, Meixing Yu

**Affiliations:** ^1^Guangzhou Women and Children's Medical Center, Guangzhou Medical University, Guangzhou 510623, China; ^2^Fudan University, Shanghai 200433, China; ^3^University of British Columbia, Vancouver, Canada

## Abstract

Autophagy plays an important role in cancer. Many studies have demonstrated that autophagy-related genes (ARGs) can act as a prognostic signature for some cancers, but little has been known in low-grade gliomas (LGG). In our study, we aimed to establish a prognostical model based on ARGs and find prognostic risk-related key genes in LGG. In the present study, a prognostic signature was constructed based on a total of 8 ARGs (MAPK8IP1, EEF2, GRID2, BIRC5, DLC1, NAMPT, GRID1, and TP73). It was revealed that the higher the risk score, the worse was the prognosis. Time-dependent ROC analysis showed that the risk score could precisely predict the prognosis of LGG patients. Additionally, four key genes (TGF*β*2, SERPING1, SERPINE1, and TIMP1) were identified and found significantly associated with OS of LGG patients. Besides, they were also discovered to be strongly related to six types of immune cells which infiltrated in LGG tumor. Taken together, the present study demonstrated the promising potential of the ARG risk score formula as an independent factor for LGG prediction. It also provided the autophagy-related signature of prognosis and potential therapeutic targets for the treatment of LGG.

## 1. Introduction

Gliomas, the primary central nervous system tumors, have four grades (I–IV) according to the descriptions of histological characteristics in the World Health Organization (WHO) [1]. Low-grade gliomas (LGG) are grade II or III tumors [2]. Primarily treated with radiotherapy, LGG patients tend to have a more favorable prognosis [3]. The median survival time for patients is between 4.7 and 9.8 years; some subtypes even have a median survival time of up to 13 years [4]. Therefore, the major target of the treatment is to maximize overall survival (OS). To further achieve this goal, it is necessary to be able to identify the high-risk patients and tailor precise treatment accordingly.

In recent years, an increasing amount of attention has been payed to autophagy, a highly conserved cellular process which is active during programmed cell death [5, 6]. It is crucial in cellular homeostasis, cancer, degenerative diseases, and organelle turnover [7]. It can act either as a tumor suppressor by means of degrading cells from damaged organelles or as a tumor-promoting mechanism for established tumors [8–10]. Studies have shown a higher efficiency in tumor killing when using the combination therapy of the late-stage autophagy inhibitor antineoplastic drugs, for instance, cisplatin and vinblastine. Hence, autophagy inhibition has been suggested to be an effective therapeutic strategy in advanced cancer [11]. Moreover, the autophagy-related gene (ARG) expression signature was constructed for independent prognosis determination in cancer patients, such as glioblastoma and kidney renal clear cell carcinoma [12, 13]. Hence, autophagy is also important in the prognosis of tumor. However, few researches are available regarding the role of autophagy in the prognosis of LGG.

To begin with, in order to establish a prognostical model based on ARGs, we collected the data of 509 LGG patients from The Cancer Genome Atlas (TCGA) and identified ARGs associated with the prognosis of LGG. Afterwards, ARGs were utilized as an independent risk factor to establish a prognosis-prediction model which was subsequently validated for LGG overall survival (OS). Furthermore, four key differentially expressed genes were identified among the high- and low-risk groups, and their associations with immune cell types which infiltrated in LGG tumor were also investigated. Results from this study suggested that autophagy-related signatures could predict the prognosis of patients with LGG, and the four key differentially expressed genes may be the candidate therapeutic targets for LGG.

## 2. Material and Methods

### 2.1. Data Collection and Immune Score Generation

The RNA-seq data of GDC TCGA Lower Grade Glioma of 509 LGG patients were downloaded from the UCSC Xena database (https://gdc.xenahubs.net), including gender, age, grade, and survival data. 232 ARGs were collected from the Human Autophagy Database (HADb, http://www.autophagy.lu/). All gene expression data were log_2_(*x* + 1) transformed and the official gene symbols were converted through gencode.v22.annotation.gene.probeMap. Immune scores were generated with the ESTIMATE algorithm using the “ESTIMATE” R package [14].

### 2.2. Building a Risk Signature Associated with the Survival of LGG Patients

The train and test sets were set up by randoming and assorting the data in the set of all the patients (509 samples) to validate the signature. Subsequently, we screened ARGs that were associated with OS of LGG patients by the univariate Cox regression analysis in the train set, and a multivariate Cox regression model was performed to further obtain selected ARGs. A risk signature was calculated based on the Coef derived from the multivariate Cox regression model coefficients and the expression value of each selected ARG. Risk score = (expr gene1 × Coef gene1) + (expr gene2 × Coef gene2) + ⋯+(expr genen × Coef genen) [15]. The prognostic associations were evaluated using the Kaplan-Meier (K-M) plots for OS based on the risk score value. Time-dependent receiver operating characteristic (ROC) analysis was also conducted to evaluate the prognosis-prediction accuracy.

### 2.3. Screening of Key Genes and Protein-Protein Interaction (PPI) Network Construction

LGG patients were separated into the high- and low-risk groups by the cutoff medians according to the value of the risk score. We compared the gene expression levels between these two groups via the “limma” package, according to the criteria of adj.*P*.Val < 0.05 and ∣logFC | >1 [16]. The 11.0 version of STRING (https://www.string-db.org) was chosen to identify the PPI network among key genes which were then visualized by the Cytoscape software (version 3.2.1). Finally, the key genes were identified using the MCODE App of the Cytoscape software.

### 2.4. Functional Enrichment Analysis

Gene Ontology (GO) functional annotation and Kyoto Encyclopedia of Genes and Genomes (KEGG) analysis with the “clusterProfiler” R package were applied to investigate the involved biological functions and pathways of the key genes. *P* value < 0.05 was chosen as the criteria.

### 2.5. Estimation of the Immune Cell Landscape

To evaluate the tumor immune infiltration levels of LGG, we utilized CIBERSORT to estimate the abundances of immune cell types via gene expression data [17]. Furthermore, we analyzed the correlations between immune cell type relative proportions and the key gene expressions with the “corrplot” package.

### 2.6. Statistical Analyses

Statistical analyses including the univariate and multivariate Cox regression models, ROC curve analysis, and K-M survival analyses were performed in the R 4.0.3 environment. The survival rates among the high- and low-risk groups were compared using the log-rank test. Differences were significant when *P* < 0.05. The heatmaps, volcano plots, and boxplots were drawn using R 4.0.3.

## 3. Results

### 3.1. Construction of a Prognosis-Prediction Model

**Figure 1:**
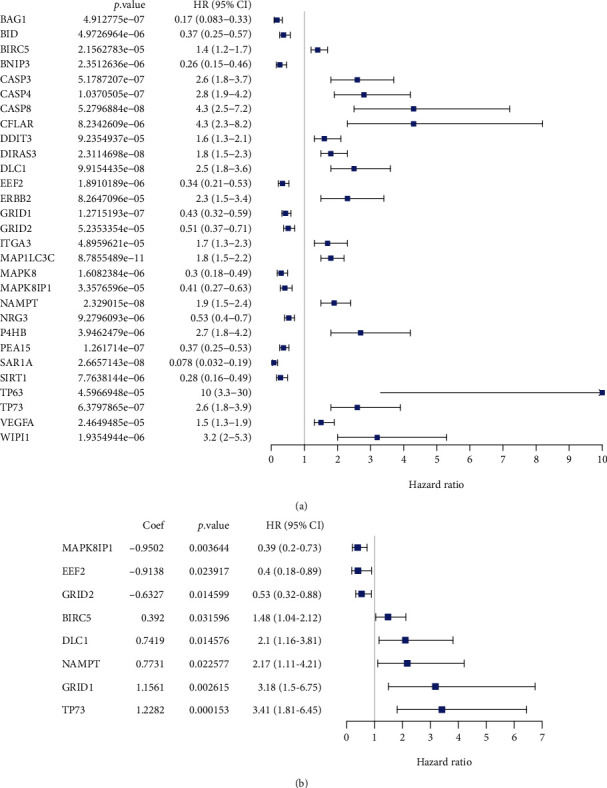
The univariate (a) and multivariate (b) regression analyses of the prognostic value for the train set. Coef: coefficient; HR: hazard ratio.

We first attained 232 ARGs from the HADb database and filtered out 509 LGG samples from TCGA. Then, the entire set (509 samples) was randomly divided into the train set (*n* = 254) and test set (*n* = 255). In the train set, we used the univariate Cox regression to screen out 29 genes (Figure 1(a)). Then, the multivariate Cox regression model was carried out, and 8 ARGs were identified (Figure 1(b)) and used to construct a risk score formula. Risk score = (−0.9502 × MAPK8IP1 expression) + (−0.9138 × EEF2 expression) + (−0.6327 × GRID2 expression) + (0.392 × BIRC5 expression) + (0.7419 × DLC1 expression) + (0.7731 × NAMPT expression) + (1.1561 × GRID1 expression) + (1.2282 × TP73 expression). It indicated that the expression levels of BIRC5, DLC1, NAMPT, GRID1, and TP73 were negatively correlated with the OS of LGG patients; however, those of MAPK8IP1, EEF2, and GRID2 were positively associated with OS.

### 3.2. The Correlation between the Risk Score and Prognosis of LGG Patients in the Train Set

**Figure 2:**
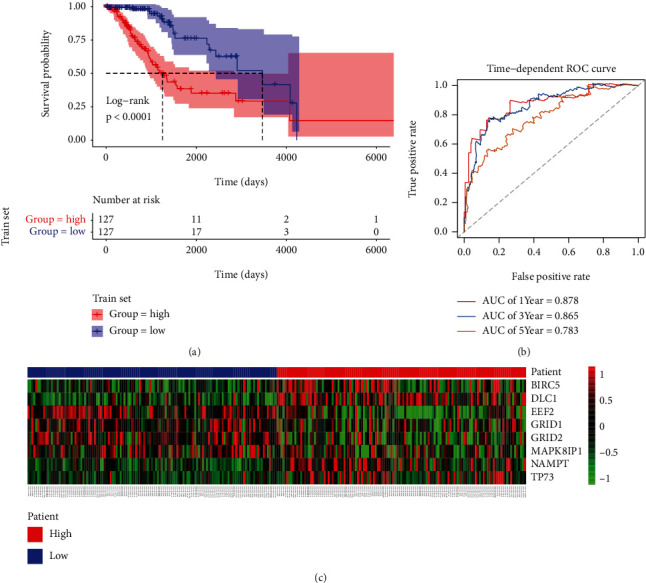
Correlation between the autophagy-related signature for OS and the prognosis of LGG patients in the train set. (a) K-M survival curves for the high- and low-risk groups. (b) ROC curves indicating the predictive accuracy of the autophagy-related signature for the 1-year, 3-year, and 5-year OS. (c) Heatmap showing expression of the 8 ARGs in the high- and low-risk groups.

To identify whether the formula could predict the prognosis of LGG patients, patients in the train set were separated into the low- (*n* = 127) and high-risk groups (*n* = 127) using the median risk score as cut-offs. Our data showed a higher survival rate in patients within the low-risk group compared to patients in the high-risk group (*P* < 0.0001, Figure 2(a)). We subsequently used a time-dependent ROC curve to determine the predictive accuracy of the risk score in prognostic prediction. The areas under the curve (AUCs) for the 1-year, 3-year, and 5-year OS of the training set were 0.878, 0.865, and 0.783, respectively (Figure 2(b)). It indicated that the risk score could provide a good prediction for LGG prognosis. Moreover, Figure 2(c) shows that the expression of BIRC5, DLC1, NAMPT, and TP73 in high-risk group increased compared to the low-risk group, while the expression levels of EEF2, GRID1, GRID2, and MAPK8IP1 were downregulated.

### 3.3. Risk Score Validation in the Test Set

**Figure 3:**
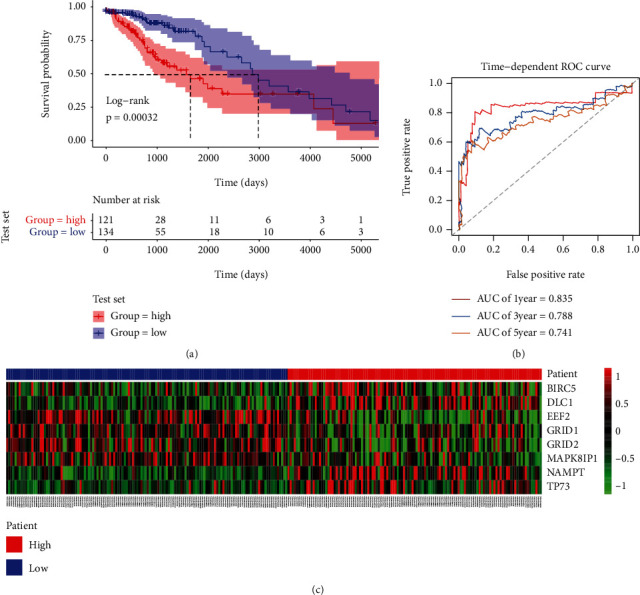
Risk score validation in the test set. (a) K-M survival curves for the high- and low-risk groups in the test set. (b) ROC curves in the test set. (c) Expression heat map.

To validate the applicability of the prognostic model, the test set was applied and separated into the low- (*n* = 121) and high-risk (*n* = 134) groups with regard to the cut-offs in the train set. Consistent with the result observed above, the OS rate of patients of the low-risk group was higher than that of the high-risk group in the test set (*P* = 0.00032, Figure 3(a)). The ROC curves also showed the good performance of the risk score in prognostic prediction: the AUCs for the 1-year, 3-year, and 5-year OS were 0.835, 0.788, and 0.741, respectively (Figure 3(b)). The expression levels of the 8 ARGs were consistent with the results in the training set (Figure 3(c)).

### 3.4. Risk Score Validation in the Entire Set

**Figure 4:**
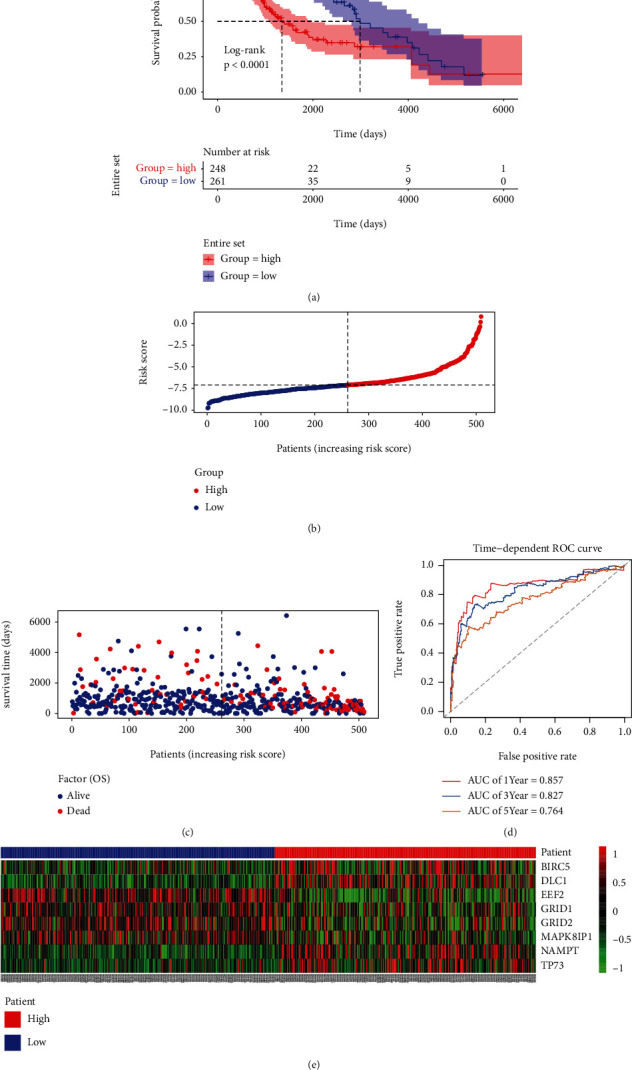
Risk score validation in the entire set. (a) K-M survival curves. (b) Rank of risk score and distribution of groups. (c) The OS of patients and distribution of groups. (d) ROC curves in the entire set. (e) Expression heat map.

Next, we verified the risk score in the entire set (*n* = 509); here, the samples were also separated into the low- (*n* = 261) and high-risk groups (*n* = 248) using the same risk score formula and cut-offs. Figures 4(b) and 4(c) display the risk score distribution and OS of patients. The K-M plot also demonstrated that low-risk patients had significantly longer OS than the high-risk patients (*P* < 0.0001, Figure 4(a)). The AUCs for the 1-year, 3-year, and 5-year OS were 0.857, 0.827, and 0.764, respectively (Figure 4(d)). As expected, the 8 ARGs' expression were also consistent with previous results (Figure 4(e)). These results indicated that the prognostic signature for OS performed well for predicting the survival of LGG patients.

### 3.5. Correlations between the Risk Score and Clinicopathologic Factors

**Figure 5:**
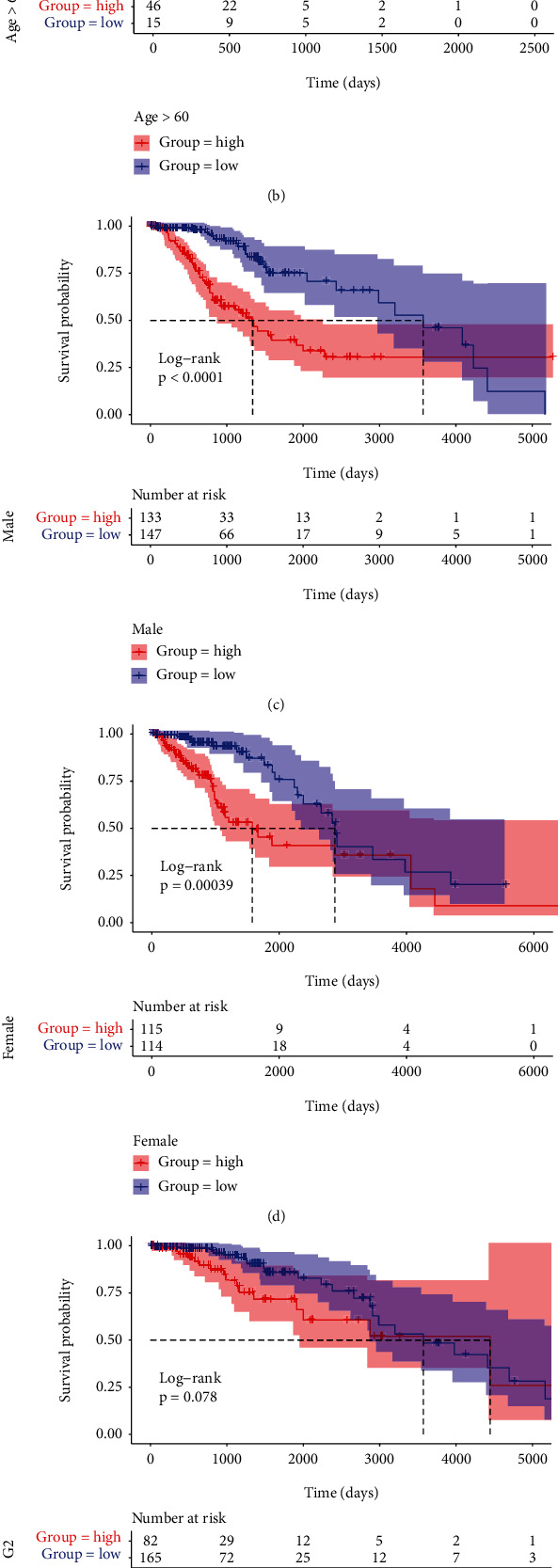
K-M survival curves for the high- and low-risk groups stratified by clinicopathological variables. (a, b) Age. (c, d) Gender. (e, f) Grade.

**Figure 6:**
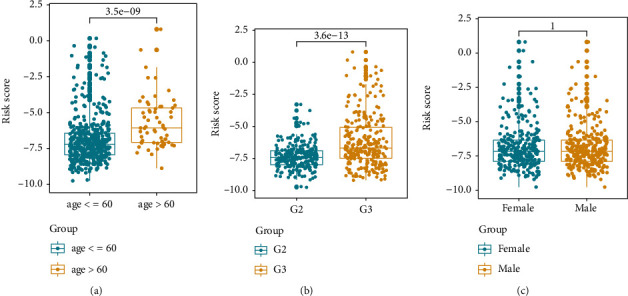
Risk score comparing between different clinicopathological factor groups. (a) Age. (b) Grade. (c) Gender.

To further understand the relationship between the risk score and clinical characteristics, we examined the prognostic value of the risk signature in different groups stratified by age, gender, and grade. However, the OS time of the low-risk group was higher than that of the high-risk group in different ages (age ≤ 60 and age > 60), gender, and grade G3 (Figure 5). No significant relation with grade G2 was observed; it could be due to the small sample size. Afterwards, risk scores were compared in some cohorts. It was demonstrated that the risk scores in the age > 60 group were obviously higher than those in the age ≤ 60 group (*P* = 3.5*e*‐09, Figure 6(a)). For grade, the risk scores increased in G3 compared with G2 (*P* = 3.6*e*‐13, Figure 6(b)). However, no statistically significant difference was discovered in risk scores between male and female (*P* > 0.05, Figure 6(c)).

### 3.6. Screening of Differentially Expressed Genes in LGG

**Figure 7:**
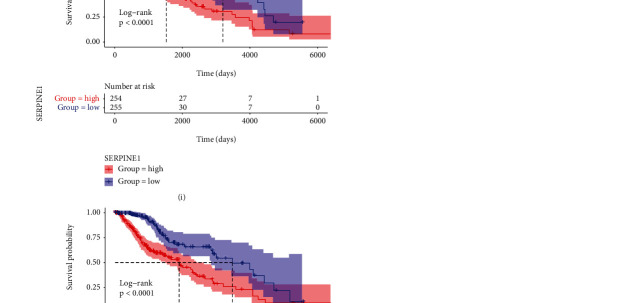
PPI network and the key gene analysis. (a) Volcano plot of the ARGs. (b) Heatmap of the 53 differentially expressed ARGs. (c) PPI network of the differentially expressed genes. (d) MCODE analysis of the differentially expressed genes. (e, f) Expression heat map of the four key genes in the high- and low-risk groups. K-M survival curves for the high- and low-risk groups stratified by the four key genes: (g) TGF*β*2; (h) SERPING1; (i) SERPINE1; (j) TIMP1. PPI: protein-protein interaction.

We compared the expression of mRNA between low-risk and high-risk samples. There were 30 upregulated and 23 downregulated genes (Figures 7(a) and 7(b)) in total. We further explored the interactive relations of these differentially expressed genes through building the PPI network using the STRING online tool and the MCODE APP of the Cytoscape software. As shown in Figures 7(c) and 7(d), TGF*β*2, SERPING1, SERPINE1, and TIMP1 are key proteins in LGG. Figures 7(e) and 7(f) show that the expression of these four key genes had higher levels in the high-risk group than in the low-risk group. Moreover, we discovered that the high expression level of each key gene was significantly associated with a worse prognosis (Figures 7(g)–7(j)). The overall results indicated that the key genes could accurately predict the prognosis of LGG patients.

### 3.7. The Correlation between Prognostic ARGs and Key Genes

**Figure 8:**
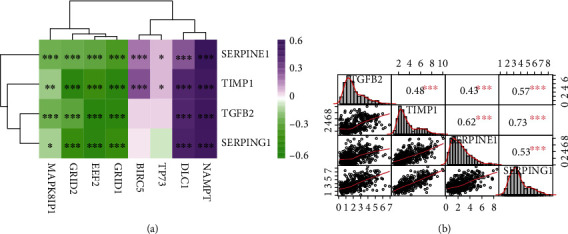
Correlation analysis. (a) Correlation between ARGs and the key genes. (b) Correlation between key genes. ^∗∗∗^*P* < 0.0001; ^∗∗^*P* < 0.01; ^∗^*P* < 0.05.

We analyzed the correlation between the 8 prognostic ARG genes and the 4 key genes (Figure 8(a)). The results indicated that 4 prognostic ARGs (EEF2, GRID1, GRID2, and MAPK8IP1) were unanimously negatively related to all key genes. Another 2 prognostic ARG genes, DLC1 and NAMPT, were positively related to all key genes. BIRC5 and TP73 were positively related to SERPINE1 and TIMP1 but were not correlated with TGF*β*2 and SERPING1. Additionally, we found that there were significant positive correlations between the 4 key genes (Figure 8(b)).

### 3.8. The Cellular Functions and Pathway Analysis of Key Genes

**Figure 9:**
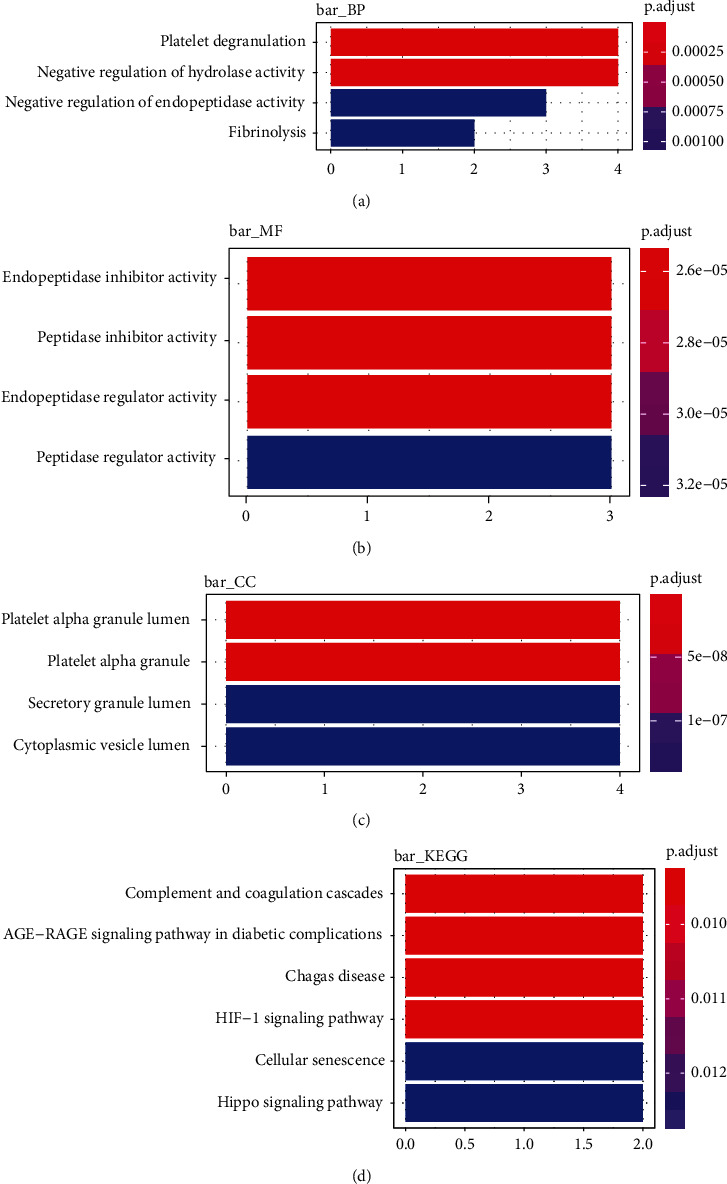
Enrichment analysis of the key genes. (a, b) GO and (c) KEGG.

To explore the cellular functions and pathways the key genes participate in, a functional enrichment analysis was conducted. It indicated that the key genes were mainly enriched in the biological process (BP) of platelet degranulation and negative regulation of hydrolase activity (Figure 9(a)) and in the molecular function (MF) of endopeptidase and peptidase-related activities (Figure 9(b)). Additionally, the main related cellular components (CCs) were platelet alpha granule lumen and platelet alpha granule (Figure 9(c)). For the KEGG pathway, the key genes were mostly enriched in complement and coagulation cascades, the AGE-GAGE signaling pathway in diabetic complications, the Chagas disease, and the HIF-1 signaling pathway (Figure 9(d)).

### 3.9. Relationships between the Immune Score, Immune Cells, and Risk Score

**Figure 10:**
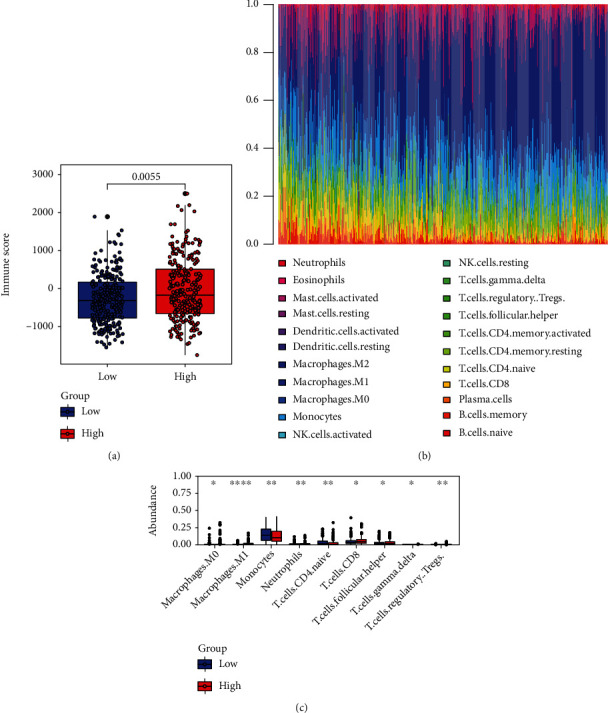
Immune scores and immune cells analysis. (a) Expression boxplots of the key genes in the high- and low-risk groups. (b) Expression heat map of the immune cell types. (c) Boxplots showing the differentially expressed immune cell types.

Considering the regulatory effect of tumor-associated platelets on tumor infiltrating immune cells and the tight correlations between autophagy and immunity, the immune score of each patient was calculated via the “ESTIMATE” package, and the values were compared in the high- and low-risk groups. The high-risk group showed a significantly higher immune score than the low-risk group (Figure 10(a)). Then, we estimated the respective proportions of 22 distinct immune cell types in LGG patients (Figure 10(b)). We found that monocytes were the predominant immune cell types. In addition, the expression of macrophages.M0 (*P* = 0.041), macrophages.M1 (*P* = 3.5*e*‐08), neutrophils (*P* = 0.0018), T.cells.CD8 (*P* = 0.025), T.cells.follicular.helper (*P* = 0.013), T.cells.gamma.delta (*P* = 0.04), and T.cells.regulatory (*P* = 0.0022) were relatively higher in the high-risk group. While the monocytes (*P* = 0.0036) and T.cells.CD4.naive (*P* = 0.0011) were lower in the high-risk group than in the low-risk group (Figure 10(c)).

### 3.10. Associations between Immune Cells and Key Genes

**Figure 11:**
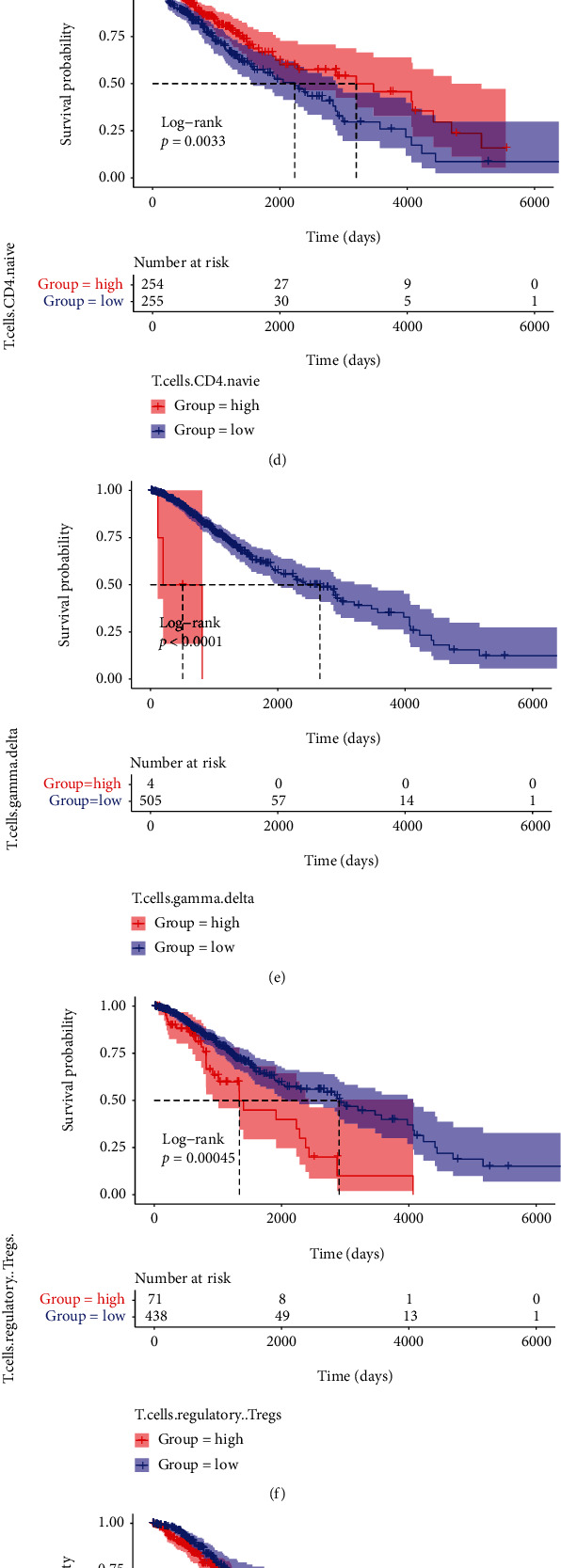
K-M survival curves for nine immune cell types. (a) Macrophages.M1. (b) Monocytes. (c) Neutrophils. (d) T.cells.CD4.naive. (e) T.cells.gamma.delta. (f) T.cells.regulatory. (g) T.cells.CD8. (h) T.cells.follicular.helper. (i) Macrophages.M0.

**Figure 12:**
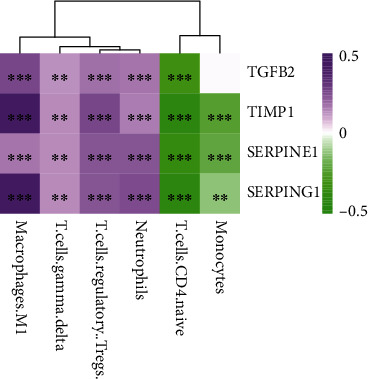
Correlation between the immune cell proportion and expression levels of the key genes. ^∗∗^*P* < 0.01; ^∗∗∗^*P* < 0.001.

We used the K-M analysis to find out whether these immune cells are related to the OS of LGG patients. It showed that a low expression of monocytes (*P* = 7*e*‐04) and T.cells.CD4.naive (*P* = 0.0033) and a high expression of macrophages.M1 (*P* = 0.036), neutrophils (*P* = 0.022), T.cells.gamma.delta (*P* < 0.0001), and T.cells.regulatory (*P* = 0.00045) were significantly associated with worse prognosis (Figure 11). Then, we analyzed the correlation between these immune cell type proportions and the expression levels of the four key genes. The results showed that the key genes were significantly associated with these immune cell types; they were positively correlated with macrophages.M1, T.cells.gamma.delta, T.cells.regulatory, and neutrophils, while they had negative relations to T.cells.CD4.naive and monocytes (Figure 12).

## 4. Discussion

Gliomas represent the most common primary brain tumor that originates from glia cells in adults [18]. LGG are indolent and slow growing WHO grade II or III primary brain tumors, and they have an overall longer survivorship than other brain tumors. Precise prognosis and therapeutic targets are crucial. In recent years, there has been an increasing amount of attention that has been payed to autophagy. Studies have reported that autophagy is associated with the occurrence and progression of cancer [15, 19, 20]. It has also been proposed that ARGs might be a useful prognostic factor for patients with glioma or/and glioblastoma [12, 21]. However, little literature is available regarding the prognostic prediction or potential prognostic biomarkers of LGG.

In this study, in addition to constructing the prognostic model for LGG, we also identified four differentially expressed genes and found the significant association between the four genes and six immune cell types (macrophages.M1, T.cells.gamma.delta, T.cells.regulatory, neutrophils, T.cells.CD4.naive, and monocytes) which infiltrated in LGG tumor. Although we did not compare LGG with normal control, we analyzed all prognostic ARGs in LGG. The ARGs we screened were more comprehensive compared with those selected by Wang et al. [22]. We further identified key differential genes related to prognostic risk and analyzed their relationship with immune infiltrating cells.

We used the univariate Cox regression to screen differentially expressed ARGs associated with the LGG prognosis, and 29 ARGs were identified. Then, via the multivariate Cox regression analysis, we identified that MAPK8IP1, EEF2, GRID2, BIRC5, DLC1, NAMPT, GRID1, and TP73 were significantly related to LGG patients' OS. Most of the 8 ARGs showed the potential of being the prognostic biomarker or therapeutic target. It has been reported that miR-10a-5p directly targets MAPK8IP1 as a major mechanism for gastric cancer metastasis [23]. Recent studies have demonstrated that EEF2 inhibited lung cancer cell proliferation, and miR-183-5p, a potential prognostic biomarker, regulates cell functions by modulating EEF2 in gastric cancer [24, 25]. This proposed role is consistent with our findings. BIRC5 is a well-known cancer therapeutic target, and its gene promoters are frequently used for transcriptional targeting of tumor cells [26, 27]. DLC1 is known to inhibit cancer progression and oncogenic autophagy in patients with hepatocellular carcinoma [28]. NAMPT is a therapeutic target in colon cancer [29]. TP73 was also reported to act as a credible biomarker for predicting favorable OS in cervical cancer patients [30].

We further constructed our prognosis model based on the above 8 ARGs. The results demonstrated that our autophagy-related signature for OS can predict the survival in patients stratified according to gender, age, and grade. The risk score was discovered to be negatively correlated with the situation of the prognosis since higher scores indicate a worse prognosis and vice versa. Our results demonstrated that the model has the potential to be converted into clinical application.

Through the PPI network and the MCODE app of the Cytoscape software, we got 4 key differentially expressed genes for further investigation by comparing the expression of mRNA between our low-risk and high-risk samples. We found that DLC1 and NAMPT were positively related to the 4 differentially expressed genes, while GRID1, EEF2, GRID2, and MAPK8IP1 were negatively related to the 4 differentially expressed genes. Furthermore, GO and KEGG analyses revealed that the four key genes were mainly enriched in platelet degranulation and endopeptidase-related activities. Granules of active platelets secrete the CD40 ligand (CD40L) so as to induce cancer cell apoptosis. While on the other hand, CD40L also improves tumor growth [31, 32]. Interestingly, the platelet degranulation was found related to the upregulation of antiautophagy genes in ovarian cancer cells [33]. Platelets release TGF*β*, which not only leads to the impairment of interferon-*γ* production and NK cell cytotoxicity but also induces the development of regulatory T (Treg) cells [34]. In addition, platelets mediate T cell suppression in cancer [35]. However, it is also believed that platelet-derived signals are required for the rapid neutrophil recruitment to form early metastatic niches [36].

Besides, the expression of each of the 4 key genes had simultaneously increased in high-risk samples. And the more each key gene is expressed, the worse is the prognosis. Our key genes were also investigated in other cancers. Studies have found that SERPINE1 is a cancer-promoting gene in gastric adenocarcinoma [37]. The lower mRNA of SERPING1 predicted lower overall survivals and higher malignancy in prostate cancer [38]. TGF*β*2 is also a valuable prognostic biomarker in gastric cancer patients [39]. TIMP1 can act as a potential prognostic indicator for colon cancer [40]. Considering their roles as mentioned above, we made further exploration to identify their prognostic signature in LGG.

Researchers have revealed the strong associations between autophagy and immunity [41, 42]. In addition, it has been reported that the immune-related gene signature was related to the prognosis in LGG [43]. We calculated the immune scores of the high- and low-risk groups. The immune scores in the high-risk group were higher than the low-risk group. CIBERSORT was used to estimate the infiltration of 22 immune cell types in the LGG samples. The result suggested that macrophages might be the predominant immune cell types which infiltrated in the LGG tumor microenvironment. In addition, we screened a total of nine types of differentially expressed immune cells in the high- and low-risk groups. Then, six types were identified that were correlated with OS of LGG patients, respectively. Furthermore, we discovered that the key genes were strongly associated with these six types of immune cells. They were positively correlated with macrophages.M1, T.cells.gamma.delta, T.cells.regulatory, and neutrophils while having negative relations to T.cells.CD4.naive and monocytes. Therefore, our results demonstrated that there might be more macrophages.M1 and less T.cells.CD4.naive which infiltrated in LGG samples than those in normal samples.

However, there are certain limitations in our study. First, the data of grade G2 and different immune cell types had relatively low sample sizes. Second, other clinicopathologic factors associated with LGG such as tumor size and metastasis stage have not been investigated. Third, we need to do further experiments to validate our findings in the future.

## 5. Conclusions

In conclusion, we constructed an autophagy-related signature based on 8 ARGs for OS that allows the prognosis of LGG patients to be accurately anticipated. Moreover, four key differentially expressed genes were found to have a significant association with OS of LGG patients and had strong relations to immune cell types which infiltrated in the LGG tumor. Therefore, the 8 ARGs may be the candidate prognostic signature for LGG, and the four key genes have a strong potential to be the novel potential targeted therapy of LGG patients. Hence, we need ongoing efforts to verify the application of the autophagy-related signature and explore the potential roles and mechanisms of key genes in LGG patients.

## Data Availability

The datasets used and/or analyzed during the current study are available from the corresponding author on reasonable request.
